# Pyroptosis in ulcerative colitis: biomarkers and therapeutic targets

**DOI:** 10.1186/s12929-025-01206-x

**Published:** 2025-12-03

**Authors:** Hannah Cruz, Prarthna Shah, Nicholas Wohlgemuth, Robert Ketchum, Imad Nassif, Chien-An A. Hu

**Affiliations:** 1 Department of Biomedical Sciences, Kansas College of Osteopathic Medicine, Kansas Health Science University, Wichita, KS 67202 USA; 2 Wichita Endoscopy Center, Wichita, KS 67226 USA; 3 Present Address: Integrated Biomedical Sciences, College of Science, Health, and Pharmacy, Roosevelt University, Schaumburg, IL60173 USA

**Keywords:** Biomarkers, Colorectal epithelial cells, Gasdermin, Inflammatory bowel disease, Pyroptosis, Therapeutic targets, Ulcerative colitis

## Abstract

**Background:**

Ulcerative colitis (UC) is one of the two major types of inflammatory bowel disease (IBD), characterized by inflammation of the colon and rectum. The colorectal epithelium, which covers the mucosal surface, maintains homeostasis by supporting commensal microorganisms in the outer mucus layer. Most colorectal epithelial cells (CECs) are absorptive colonocytes distributed primarily in the upper portion of the crypts. These CECs constitute the front-line barrier that modulates mucosal immunity and facilitates the transfer of immune molecules into the lumen. In patients with UC, CECs undergo both apoptosis and pyroptosis. Apoptosis is a physiological, programmed, caspase-dependent, and tightly regulated form of cell death that eliminates aged and damaged cells. In contrast, pyroptosis is an inflammatory, caspase-dependent form of lytic cell death that occurs in response to harmful stressors and toxins. Pyroptosis in CECs involves a broad array of signaling and effector molecules, many of which serve as measurable biomarkers with diagnostic, prognostic, and therapeutic potential.

**Conclusions:**

Dysregulated colorectal microflora significantly contributes to activating the pyroptotic pathway, initiating an inflammasome- and gasdermin-dependent inflammatory cell death process in UC patients. This review discusses the triggers and pathways of pyroptosis in CECs, evaluates recently identified biomarkers, highlights their potential roles in pyroptosis and as therapeutic targets in managing UC, and candidate compounds that have been shown effective UC therapeutics.

## Background

The colonic epithelium (CE) covers the colonic mucosal surface and maintains intestinal homeostasis by harboring the commensal microorganisms in the outer mucus layer. The majority of colonic epithelial cells (CECs) are absorptive colonocytes distributed in the upper portion of the crypt. These cells not only coordinate absorption and secretion within the digestive tract but also modulate the barrier immunity and transfer immune molecules to the lumen that are essential for host defense and immune tolerance. Dysregulation of colonic homeostasis is frequently associated with inflammation, which underlies various disorders and diseases, including bacterial infection, inflammatory bowel disease (IBD), and colorectal cancer.

Ulcerative Colitis (UC) and Crohn’s disease (CD), the two major types of IBD, are diagnosed through endoscopy, imaging studies, or biopsy in a patient exhibiting relevant clinical signs and symptoms. Although UC and CD both cause inflammation in the digestive system, they exhibit significant clinical distinctions. While CD can affect any segment of the digestive tract, UC exclusively affects the colon and rectum. UC typically manifests with mucosal inflammation and cell death, characterized by irregular periods of remission and relapse. The global *per capita* annual incidence of UC is approximately 9–20 cases per 100,000 and has been steadily increasing, particularly in Western developed countries. Consequently, UC imposes substantial health, social, and economic burdens worldwide, significantly reducing patients’ quality of life [1, 2].

Several risk factors for the development of UC have been identified, including genetics, environmental factors, innate immunity, and the colonic microbiota. Symptoms of an active UC flare include bloody diarrhea, rectal urgency, tenesmus, and abdominal pain that is relieved with defecation. A defining characteristic of UC is continuous colonic inflammation, which can lead to erythema, loss of vascular pattern, granularity, erosions, friability, bleeding, and ulcerations. Furthermore, patients with UC often exhibit decreased crypt density, distorted crypt architecture, an irregular mucosal surface, and severe diffuse transmucosal inflammation. Typically, histopathologic evaluation is employed to establish a definitive diagnosis of UC, based on abnormal histological findings, including those described above. Additionally, patients with UC have a mean colon wall thickness of 8 mm, which significantly differs from the mean colon wall thickness of 2–3 mm in unaffected individuals [1–3].

Co-morbidities are commonly associated with UC, including impaired social interactions, psychological disorders such as anxiety and depression, epithelial dysplasia, and colorectal cancer [1, 2]. Notably, patients with UC residing in Asia exhibit a significantly elevated risk of developing colorectal cancer within a shorter time frame (10–20 years of disease duration) compared to individuals with UC living in North America (30 years of disease duration) [4, 5]. This suggests that additional factors beyond genetics, such as the gut microbiota and dietary patterns, may also be significant contributors.

Due to variations in living conditions, dietary habits, and lifestyles, the colonic microbiota of individuals from different geographical locations exhibits substantial variability, potentially playing a role in UC etiology [5]. Given the prevalence, severity, and morbidities of UC, it is crucial to develop appropriate diagnosis and treatment paradigms that can provide relief and comfort to patients while mitigating the risks for the development of severe complications, such as colorectal cancer.

Pyroptosis, a highly inflammatory form of programmed cell death, is widely believed to significantly contribute to the pathogenesis of UC and the progression to colitis-associated colorectal cancer. Pyroptosis amplifies the release of pro-inflammatory cytokines and damage-associated molecular patterns (DAMPs), which can perpetuate chronic mucosal injury and immune activation. Although this plausible link exists, the precise role of pyroptosis in initiating or accelerating colitis and colitis-associated colorectal cancer in humans remains incompletely elucidated. Therefore, further mechanistic and translational studies are necessary to clarify its contribution [6].

Our literature review conducted extensive searches for articles pertaining to ulcerative colitis (UC) and pyroptosis, with the objective of identifying biomarkers directly or indirectly associated with the pyroptotic pathway. These biomarkers hold potential for utilization in the diagnosis and treatment of UC. By elucidating these biomarkers, we aspire to provide diagnostic and treatment options for individuals affected by UC.

## Main text

### Regulated cell death (RCD) in the intestinal epithelium

Cellular homeostasis is maintained through the dynamic equilibrium between cell death and survival. UC research continues to prioritize the understanding of the diverse cell death mechanisms pertinent to intestinal physiology and pathophysiology. Within the intestinal epithelium, four regulated cell death pathways have been extensively studied: apoptosis, pyroptosis, necroptosis, and ferroptosis. Apoptosis and pyroptosis represent the primary, interactive, physiological pathways that influence the pathogenesis of UC [7, 8]. Both apoptosis and pyroptosis serve as mechanisms to prevent and counteract necrosis. Necrosis, a non-programmed, highly immunogenic, and pathological cell death, is characterized by its acute and unregulated nature. It leads to the disruption of the plasma membrane, resulting in the leakage of cellular contents into the extracellular space. This process triggers acute and systemic immune responses [8].

**Fig. 1:**
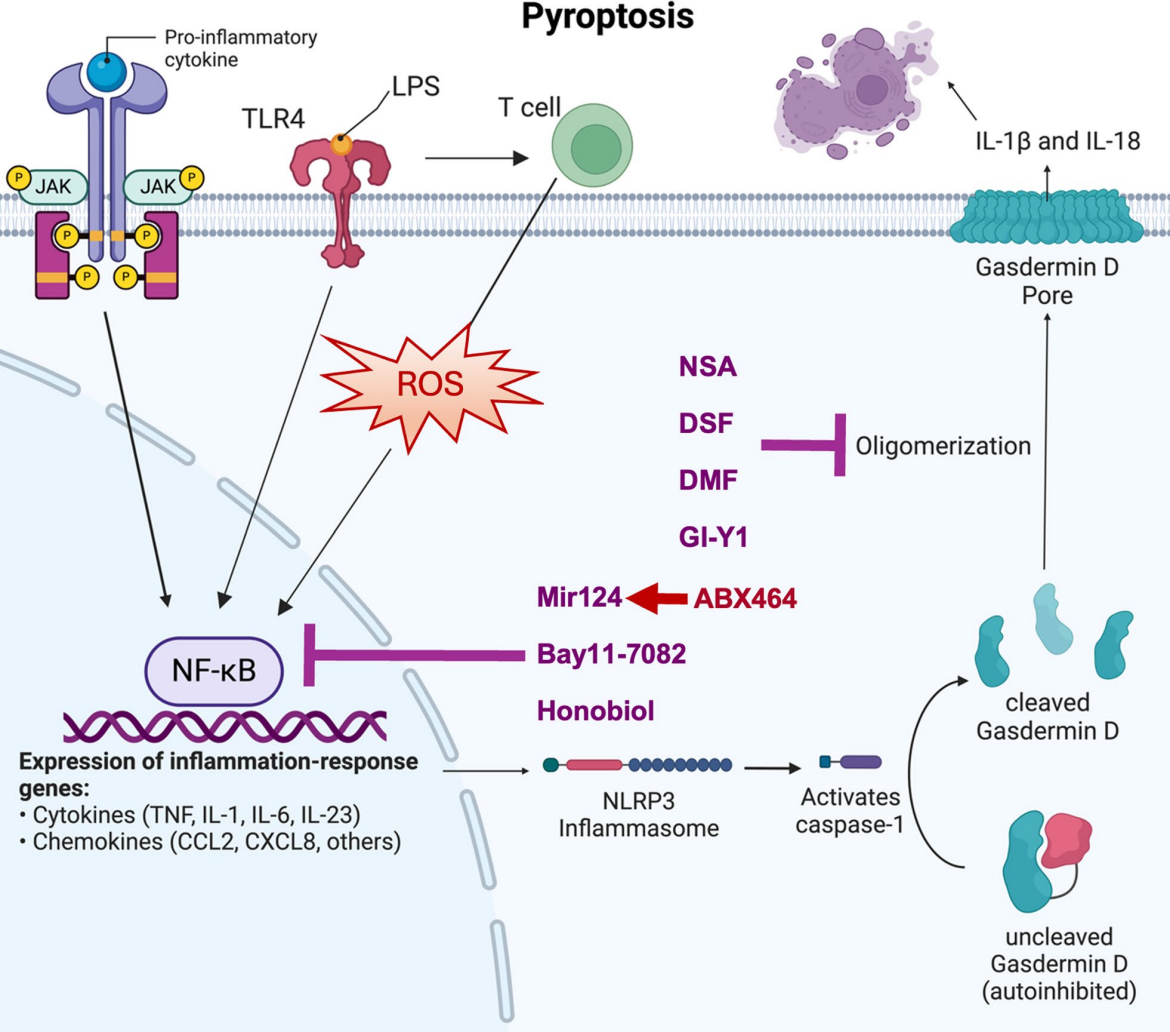
Epithelial Pyroptosis in UC. Pyroptosis is commonly initiated by extracellular stressors, for example, inflammatory cytokines, lipopolysaccharide (LPS) released by lumenal bacteria, and T cells acting on colorectal epithelial cells (CECs). A pro -inflammatory cytokine, once activated, can in turn, activate the JAK/STAT pathway. The JAK/STAT pathway is a key component of the signaling process in inflammation, especially in UC. JAK proteins induce a downstream effect of activating STAT, which is an activator of transcriptional processes. This in turn leads to many downstream processes of inflammation. LPS induces inflammation through the stimulation of toll-like receptor 4 (TLR4) on the surface of CECs, resulting in an intracellular response that recruits specific transcription factors such as NF-kB. NF-kB turns of the expression of cytokines (for example, TNF, IL-1, IL-6, and IL-23) and chemokines (for example, CCL2, CXCL8). On the other hand, T cell can induce the production of intracellular reactive oxygen species (ROS) that activates NF-κB. Consequently, intracellular cytokines and chemokines trigger the formation and activation of inflammasome (for example, NLRP3), leading to the activation of inflammatory caspases, such as caspase-1. Activated caspase-1 then cleaves and activates GSDM, for example, GSDMD, resulting in activated C-terminus GSDMD to form plasma membrane pores and release pro-inflammatory molecules, such as IL-1beta, and IL-18. Compounds, such as NSA, DSM, DMF, and GI-Y1, can block GSDM oligomerization and pore formation, whereas Mir-124, Bay11-7028, and honobiol can block NF-kB-medicated inflammatory response. ABX464 is an inducer of miR-124 expression

Pyroptosis is a gasdermin (GSDM)-dependent form of cell death that shares several characteristics with apoptosis, including a caspase-dependent pathway and the involvement of damaging condensation of the nuclear chromatin and DNA. The primary distinction lies in the pro-inflammatory and oxidative nature of pyroptosis [8]. Pyroptosis is commonly initiated by extracellular stressors that trigger the formation and activation of inflammasomes, such as NLRP3. This activation leads to the activation of inflammatory caspases, including caspases-1, 4, 5, and 11. Activated caspase-1 then cleaves and activates GSDMD, resulting in the formation of an activated N-terminus GSDMD that creates plasma membrane pores. These pores release pro-inflammatory molecules, such as IL-1β and IL-18 [8, 9] (Fig. 1). The inflammasome was discovered in 2002 by Tschopp et al. They described “an inducible high molecular weight complex containing NALP1, Pycard, and proinflammatory caspases,” which they termed an “inflammasome.” [10] They found that various microbial agents, such as bacterial lipopolysaccharides (LPS), induced the activation of inflammasomes in immune cells, leading to the activation of caspase-1 and caspase-5, both of which further induce IL-1β activation downstream and produce an inflammatory response. NALP1 was found to be a crucial nucleotide binding site (NBS), which acts as a sensor region on the inflammasome complex to detect signals such as LPS to induce activation of inflammasomes. The importance of inflammasomes in regard to UC comes from its connection to autoimmune and inflammatory diseases; the improper or overactivation of inflammasomes may result in excessive inflammation, a hallmark of IBDs, including UC [10].

The discovery that GSDMD was cleaved by caspase-1 came in 2015. Using CRISPR-Cas9 screening from mouse bone marrow macrophages, genes involved in pyroptosis were investigated. It was found that cells lacking GSDMD resisted the induction of pyroptosis despite the presence of LPS, inflammasomes, and Caspase-1. This finding demonstrated the importance of GSDMD in the activation of pyroptotic cell death. Further investigation found that Caspase-1 cleaved a region of GSDMD between its N-terminal (Gasdermin-N) and C-terminal domains, which removed the autoinhibitory effect of the C-terminal region and freed Gasdermin-N to drive pyroptosis [11].

The connection between pyroptosis and IBD was first made in 2015. This link was made by analyzing genetic loci associated with IBD, which found that decreased expression of GSDMB and increased expression of GSDMA in intestinal tissue biopsies were correlated with IBD. Given the role that GSDMD plays in the pyroptotic pathway and the gasdermin family link between GSDMD and GSDMA/B, a connection between IBD and pyroptosis was suspected. Further investigation found that GSDMA induced apoptosis while GSDMB encouraged epithelial cell proliferation in the intestinal epithelium. Given the imbalance in GSDMA/B in intestinal tissue biopsies with IBD, it was proposed that this dysregulation resulted in pyroptotic cell death without adequate tissue regeneration, leading to chronic intestinal inflammation [12].

Using animal models and transcriptomic analyses, Zhao et al. [13] demonstrated that pyroptosis-related signature genes are upregulated in UC, contributing to colonic inflammation and mucosal injury. Although these findings are not directly applicable to humans, they provide a direction for future research investigating the impact of pyroptosis in individuals with UC. Furthermore, given that dysbiosis and enteropathogenic bacterial toxins can activate inflammasomes in the gut, Yang et al. [14] showed that the immune activation in UC likely induces pyroptosis in colonic epithelial cells, contributing to the mucosal inflammation and tissue damage characteristic of the disease.

The involvement of specific biomarkers, such as IL-1β, is implicated in the pyroptotic pathways involved in UC through the activation of NLRP3. Animal models have investigated this pathway’s involvement in UC by comparing wild-type mice to NLRP3 knockout mice. The study revealed significantly less colitis and mortality in mice lacking NLRP3 gene expression; however, close and frequent exposure to wild-type mice led to susceptibility of the knockout mice to colitis. These findings suggest that the transmission of bacterial flora between the two strains of mice may be a contributing factor to UC [15]. Human studies have yielded similar results regarding the involvement of NLRP3 in UC. For example, Mortlock et al. [16] reported that polymorphisms in genes such as NLRP3 and CARD8 are linked to variations in inflammasome activity and cytokine release, which may help explain differences in disease progression and treatment response among individuals. Investigating the molecular basis of this heterogeneity could inform personalized and precision therapeutic strategies for UC.

While microbial triggers, such as LPS, are well-characterized activators of pyroptosis, other non-microbial factors have also been implicated in inflammasome activation in UC. Mitochondrial dysfunction elevates reactive oxygen species (ROS) levels, which activate the NLRP3 inflammasome and enhance the secretion of IL-1β and IL-18 [17]. Heavy metals and pollutants have been demonstrated to induce oxidative stress and disrupt epithelial barrier integrity, sensitizing cells to inflammasome activation [18]. Saturated fatty acids and emulsifiers can compromise tight junctions, leading to increased intestinal permeability and facilitating microbial translocation, which subsequently activates the inflammasome [19].

The direct relationship between genetic variants, their functional impact on pyroptosis, and treatment response in UC is an area of active investigation. Genetic heterogeneity, such as variants in loci, such as HLA-DQA1, HLA-DQB1, and CFB, has been shown to be associated with disease severity. However, no specific variants have been reported to functionally alter pyroptotic pathways in human tissue [16]. Interestingly, transcriptomic and bioinformatic analyses of human UC samples have identified genes related to pyroptosis, such as AIM2, CASP1, CASP5, GSDMD, IL1B, and TLR4, which were differentially expressed in UC patients. The protein products encoded by those genes play important roles in inflammasome activation and pyroptosis execution [13, 20, 21]. In addition, studies employing human UC tissue and animal models demonstrated a correlation between levels of expression of pyroptosis-related genes and disease activity and mucosal inflammation. This evidence supports a functional role for pyroptosis in UC pathology. Regarding treatment response, bioinformatic analyses of UC patient data have indicated that a higher pyroptosis burden, characterized by elevated expression of genes such as AIM2 and CASP5, is associated with a diminished response to anti-tumor necrosis factor (TNF) therapy. Mechanistically, increased pyroptosis can perpetuate mucosal inflammation and compromise the integrity of the epithelial barrier. This may diminish the efficacy of anti-TNF therapies, as these agents primarily target downstream TNF signaling rather than upstream inflammasome activation [21].

### Microflora, dysbiosis, pyroptosis, and inflammatory response in ulcerative colitis

The immune response of the intestinal tract is regulated by a barrier layer of colonic microbiota, which plays a crucial role in preserving the intestinal barrier integrity. Integral to the preservation of this barrier between the gut’s microflora and the colon are the CECs [22]. Under normal circumstances, specific inflammasomes within the CECs are activated to ensure colonic homeostasis. However, disruption of this protective barrier can potentially result in dysbiosis [23]. A compelling association between the dysregulation of colonic microbiota, the compromised mucosal immunity, and the aggregated intestinal inflammation in patients with UC has been demonstrated. The gut microbiome comprises a diverse array of bacteria, encompassing anaerobic, facultative anaerobic, and aerobic species. Anaerobic bacteria constitute a substantial portion of the gut microbiota in the lower portions of the intestinal tract, where they fulfill symbiotic functions, such as aiding in digestion and nutritional regulation [24]. Certain bacteria living within the host demonstrate conditional characteristics, remaining harmless under normal circumstances but becoming detrimental in extreme or altered environments. In the case of UC patients, an imbalance in colonic microflora precipitates the proliferation of opportunistic and foreign pathogens in the colon and/or rectum. This perturbed environment elicits inflammation and fosters the proliferation of deleterious pathobionts, contributing to the diseased state [22–24]. Numerous studies have identified specific pathogens, such as *Akkermansia muciniphila*, which potentially contribute to the inflammatory processes observed in UC patients [24, 25]. The intestinal microbiome has demonstrated modulatory influence over pyroptotic cell death within CECs. Microbial components such as LPS and other pathogen-associated molecular patterns can activate pattern recognition receptors (e.g., TLR4, NLRP3) on CECs, triggering the NF-κB/NLRP3/GSDM signaling axis and resulting in pyroptosis with release of pro-inflammatory cytokines IL-1β and IL-18. Additionally, certain microbial metabolites like butyrate can suppress IEC pyroptosis by inhibiting the cGAS-STING-NLRP3 pathway, highlighting the complex regulatory role of the microbiome in modulating epithelial cell death and intestinal inflammation [26, 27].

### Diagnostic methods for UC

The diagnosis of UC can be approached through various methods. Ileocolonoscopy and biopsy analysis are considered the most definitive for evaluating the extent and severity of inflammation. Endoscopic imaging provides detailed visualization of mucosal characteristics, such as granular or fragile mucosa, alterations in vasculature patterns, and the presence of abnormal tissue growth like polyps [28].

While endoscopy and radiographic modalities can be helpful in diagnosing UC, they are not indispensable. Given that UC can affect various regions of the colorectal area, including the rectum, sigmoid colon, or the entire colon, a laboratory test is necessary to measure biomarkers that can establish a definitive diagnosis [29]. Biomarkers are quantifiable molecules indicative of disorder or infection and are used for diagnostic, prognostic, and therapeutic purposes. A comprehensive analysis of biomarkers in UC patients should include pro-inflammatory molecules, key pyroptosis effectors, and colonic microbiota [30]. Some biomarkers intimately related to the etiology of UC are as follows:

#### Proprotein convertase subtilisin kexin type 9 (PCSK)

PCSK9, a proprotein convertase and pro-inflammatory enzyme, plays a crucial role in the development and progression of IBD. It influences inflammation by inducing cytokines such as IL-1β, IL-6, and TNF, as well as pro-inflammatory chemokines. Wang et al. [31] showed that PCSK9 contributes to the pathogenesis of low-grade, atherosclerosis-related inflammation and cardiovascular disease, and triggers pyroptosis in myocardial infarction, which is associated with mitochondrial damage. Furthermore, Yang et al. [32] showed that PCSK9 activation in mice reduces antioxidant pathways, contributing to inflammation through lipid hydroperoxide buildup. Conversely, PCSK9 downregulation lowers the secretion of pro-inflammatory factors. Moreover, Marinelli et al. [33] showed that elevated levels of PCSK9 were observed in blood tests of UC patients during flares, suggesting the involvement of a pyroptotic pathway in UC. When compared with 112 UC patients with healthy controls, a positive correlation between PCSK9 levels and abnormal fecal calprotectin (FCP) and/or C-reactive protein (CRP) was found. Higher endoscopic scores in UC were also associated with greater PCSK9 levels. Thus, the study demonstrated a positive correlation between PCSK9 levels and FCP, endoscopic Mayo Score, UC-Riley Index, and both total and low-density lipoprotein cholesterol values. It was concluded that serum PCSK9 levels are increased in patients with biochemical and endoscopic evidence of active disease in UC (Table 1).

**TABLE 1:** Representative biomarkers in ulcerative colitis

Biomarker	Functions in ulcerative colitis	Functions in pyroptosis	Response to inflammation	References
PCSK9	Correlates with endoscopic and biochemical disease activity	Primarily involved in lipid metabolism & inflammation modulation	↑ in active UC	[33]
IL-33	Acts as an alarmin: released by stressed epithelial cells; elevated in active UC mucosa (dual pro/anti‑inflammatory roles); serum levels ↑ in active pediatric UC	Released downstream of GSDM-dependent pyroptosis	↑ in inflamed intestinal mucosa; serum levels ↑ in active UC (may decline with resolution)	[36, 37]
CRP	Acute-phase reactant: widely used to monitor UC activity	Stimulated simultaneously with GSDME by IL-6, which activates the pyroptotic pathway	↑ in active UC; ↑ in deep tissue ulcers; high serum levels associated with ↑ UC disease severity	[39–41]
SAA	Another acute-phase protein; increases during systemic inflammation	Stimulated simultaneously with GSDME by IL-6, which activates the pyroptotic pathway	↑ in active UC; correlates with disease severity	[42]
CP	Neutrophil-derived protein; excellent marker of mucosal inflammation in UC	DAMPs released during pyroptosis trigger the release of inflammatory proteins, including FCP	↑ in active UC; used to monitor flares/remission	[43, 45]
GSDM	Induce inflammatory mechanisms in colonic epithelial cells; elevated levels have been found in human intestinal epithelial cells in UC	Cleaved by inflammatory caspases (for example, caspase− 1/− 11), forming lytic pores that allow for the release of inflammatory markers, such as IL-1β and IL-18, and proptosis	↑ in active UC; promotes inflammation through release of inflammatory cytokines	[49, 50, 52]

PCSK9, Proprotein convertase subtilisin kexin type 9; IL-33, Interleukin-33; CRP, C-reactive protein; SAA, Serum amyloid A; CP, calprotectin; GSDM, Gasdermin

#### Interleukin-18 (IL-18)

IL-18 is a crucial cytokine involved in the pathogenesis of colitis, playing both pro-inflammatory and anti-inflammatory roles depending on the stage and context of the disease. Its ability to promote inflammation and goblet cell dysfunction, as well as its involvement in driving mucosal barrier breakdown, suggests that targeting IL-18 could be a valuable approach for treating colitis [34, 35]. 1

#### Interleukin-33 (IL-33)

IL-33, a pleiotropic cytokine, exhibits dual functions as an extracellular cytokine and a nuclear transcription factor. Studies have demonstrated that IL-33 expression is elevated in active lesions of UC patients compared to inactive lesions and even active lesions of Crohn’s disease patients, underscoring its potential as a biomarker for UC [36, 37]. Furthermore, an imbalance in the IL-33 suppression of the tumorigenicity 2 (ST2) axis has been observed in the colonic mucosa of UC patients [37]. ST2, a receptor for IL-33, regulates both innate and adaptive immunity in inflammatory disorders. The IL-33/ST2 axis has been implicated in maintaining intestinal homeostasis. Notably, IL-33 has been identified as one of the inflammatory cytokines released through pyroptosis in a GSDMD-dependent manner [38]. Given its role as a pro- or anti-inflammatory mediator of first-line innate immunity and pyroptosis, the levels of IL-33 and ST2 in colonic tissue and blood samples are crucial in defining mucosal defenses and cell death (Table 1).

#### C-reactive protein (CRP)

CRP is a pentameric protein synthesized in the liver and circulating in the bloodstream. Elevated CRP levels indicate inflammation. CRP plays a crucial role in innate immunity, safeguarding the body and regulating host defense responses to tissue injury. It is a soluble, non-glycosylated, and non-covalently associated protein composed of five identical globular subunits. High CRP levels are associated with tissue damage caused by inflammatory processes. CRP is stimulated by the cytokine IL-6, which is released by macrophages, endothelial cells, and epithelial cells that have experienced damage. IL-6 simultaneously activates the GSDM-mediated cell death pathway, underscoring CRP’s potential as a diagnostic biomarker for pyroptotic cell death. CRP can undergo rapid fluctuations in serum levels, increasing up to 100-fold within 72 h [39]. Given its efficient response to tissue damage and inflammation, serum CRP serves as a valuable biomarker in the diagnosis of UC.

Several isoforms of CRP exist, each with distinct regulatory functions regarding the inflammatory response. Pentameric CRP (pCRP), the isoform commonly measured in blood, exhibits weak anti-inflammatory effects. Conversely, monomeric CRP (mCRP) exhibits strong pro-inflammatory impacts on tissues. Notably, pCRP serves as a precursor to mCRP, undergoing conversion to the mCRP isoform in response to tissue damage.

Given pCRP’s prevalence as a biomarker for inflammation analysis, interpreting serum CRP levels can be intricate. The conversion of pCRP to mCRP occurs rapidly upon tissue damage, but this process decelerates over time, resulting in an accumulation of pCRP in the bloodstream. Consequently, elevated pCRP levels are indicative of chronic inflammation, making them a diagnostic marker for inflammatory diseases such as UC [40]. Subsequently, Grant et al. [41] investigated the impact of CRP levels on treatments for UC. Notably, in 78.1% of individuals with a CRP level exceeding 50 mg/L, intravenous steroid therapy failed to elicit a response to the inflammatory process of UC. This observation suggests a correlation between high CRP levels and the severity of UC disease, necessitating alternative or additional treatments to alleviate UC symptoms (Table 1).

#### Serum Amyloid A (SAA)

Similar to CRP, SAA is an acute-phase protein, and its increased levels are linked with inflammatory conditions such as UC and CD. It can stimulate the release of pro-inflammatory cytokines like IL-1β, IL-18, and TNF-α. SAA expression is significantly higher in the intestinal tracts of UC**.** Frequent and costly endoscopic procedures may be challenging for patients, prompting a comparison of SAA to CRP as a cost-effective biomarker for mucosal healing in UC. Wakai et al. [42] showed that SAA exhibited higher specificity and sensitivity as a marker of inflammation compared to CRP, extending beyond IBD. The group used the Mayo endoscopic subscore (MES) to assess mucosal inflammation, with MES zero indicating complete healing, one indicating healing, and scores of two or three indicating non-mucosal healings. They found a higher correlation between SAA levels and MES scores compared to CRP and MES scores. This finding, combined with the previously noted correlations between CRP levels and severity of UC disease, highlights SAA’s value as a potential biomarker of mucosal healing diagnosis in UC patients [42] (Table 1).

#### Calprotectin (CP)

Fecal calprotectin (FCP), a prominent neutrophil protein, is commonly employed as a nonspecific biomarker for UC due to its stability. Neutrophil migration and recruitment initiate a cascade that leads to the release of reactive oxygen species, enhanced respiratory burst, and FCP secretion [43]. In pyroptosis, DAMPs are released, triggering an inflammatory response through their downstream effects, including the release of FCP [44]. Calprotectin levels exhibit a linear correlation with neutrophil counts, indicating that elevated FCP levels are associated with inflammation severity, particularly in UC patients. Consequently, FCP has been identified as a predictive UC biomarker. Heinzel et al. [45] showed that while FCP exhibits high sensitivity in identifying UC disease, its specificity is low, as levels can be significantly elevated during infections unrelated to IBD. Furthermore, a meta-analysis done by Walsham et al. [43] reported that FCP is a reliable diagnostic tool for distinguishing patients with IBD from those with irritable bowel syndrome, with pooled sensitivity and specificity values of 93% and 96%, respectively. These findings suggest that while FCP serves as a valuable marker for intestinal inflammation, its elevation is not exclusive to pyroptosis in UC and can result from various inflammatory processes. Therefore, interpreting FCP levels should encompass the broader context of intestinal inflammation (Table 1).

#### Fecal lactoferrin (FL)

FL, a glycoprotein, is expressed by activated neutrophils during periods of trauma and inflammation, particularly in patients with UC and Crohn’s disease (CD) [46, 47]. Numerous studies have been conducted to assess and validate the specificity and sensitivity of FL in patients with mucosal inflammation. Similar to C5a, elevated FL levels accurately indicate intestinal mucosal inflammation. Kane et al. [46] demonstrated that FL levels exhibited 90% specificity in identifying inflammation in active IBD patients and 100% specificity in ruling out IBD. Consequently, FL may prove beneficial in screening for inflammation in patients presenting with digestive disorders [47]. Other studies have indicated a strong correlation between FL levels and disease severity and mucosal inflammation. For example, Rubio et al. [48] showed that FL can accurately and promptly represent intestinal inflammation in IBD patients. However, while elevated FL levels are associated with inflammation, their direct connection to pyroptosis requires further investigation. The specificity of FL as a biomarker exclusively for pyroptosis-driven inflammation has not been definitively established. Therefore, additional validation through larger, multicenter studies is necessary to fully elucidate its role and potential as a biomarker for pyroptosis in the context of IBD.  1

#### Gasdermin (GSDM)

GSDM proteins are essential components of the pyroptotic pathway. Upon activation, GSDMs undergo cleavage by caspases-1 and -11, facilitating the release of IL-1β and IL-18. This mechanism triggers inflammatory responses in human cells [49] (Fig. 1). There are several GSDM isoforms present in different cell types, with GSDME being particularly implicated in various inflammatory diseases. When activated, GSDME forms lytic pores, inducing pyroptosis [50]. GSDMD, another GSMD isoform, has been observed to be elevated in human inflammatory intestinal tissues. GSDMD differs from GSDME in its activation mechanism and is associated with both apoptosis and pyroptosis in IBDs [51]. Like GSDME, GSDMD forms pores in cell membranes, inducing pyroptosis and damaging the epithelial lining of the bowel [52] (Table 1).

### Therapeutic targets in treating UC

The pathogenesis of UC encompasses host genetic factors, commensal microbiota, and the immuno-inflammatory pathways in the colonic mucosa. Several components in the colonic epithelium, including the epithelial barrier, antigen recognition, immunological responses, T cell and B cell recruitment, and pyroptosis, are directly involved in the etiology of UC. Clinically, IL-23 and TNF-α are two current targets for UC treatments. IL-23 is a key driver of UC pathogenesis, promoting inflammation by supporting Th17 cell differentiation and cytokine production [53]. Its elevated expression in colonic tissue correlates with disease severity, and genetic variations in the IL-23 receptor (IL23R) have been linked to UC susceptibility. Using preclinical models, Sewell and Kaser [54] demonstrated that blocking IL-23 reduces intestinal inflammation, highlighting its therapeutic potential. IL-23 inhibitors, such as ustekinumab and risankizumab, have shown effectiveness in clinical trials for inducing and maintaining remission in UC, reinforcing IL-23 as a promising target for treatment. TNF-α is a crucial cytokine in UC, driving inflammation by activating immune cells and hindering the repair of intestinal tissue. Its elevated presence in UC patients makes it an important therapeutic target. Anti-TNF therapies such as infliximab, adalimumab, and golimumab work by neutralizing TNF-α, reducing inflammation, and aiding in mucosal healing, with demonstrated effectiveness in inducing and maintaining remission [55]. However, not all patients respond to these treatments, and some may experience a loss of efficacy over time, underscoring the need for better predictive markers and a deeper understanding of TNF-α’s role in UC. Approximately 30% of patients with IBD exhibit primary nonresponse to anti-TNF-α therapies, and 23% to 50% of those who initially respond experience a loss of response during ongoing treatment [56].

Therapeutic targets in patients with UC are also indicated by the levels of various biomarkers. It has been extensively demonstrated that PCSK9, CP, and GSDMD not only serve as biomarkers but also constitute therapeutic targets in UC. For instance, PCSK9, a pro-inflammatory enzyme, plays a pivotal role in the inflammatory process of UC. Inhibiting PCSK9 effectively reduces the inflammatory response, as evidenced by the elevated serum PCSK9 levels commonly observed during UC flares [33]. FCP, the primary protein component of neutrophils, exhibits a comparable response in these patients. Elevated CP levels signify the activation of the inflammatory response mediated by cytokines and released neutrophils in diseased patients. Inhibiting factors of CP presented to neutrophils diminish the release of inflammatory cytokines, resulting in a reduced presence of these leukocytes in the feces [57].

### Compounds target GSDM polymerization and pore formation

GSDM proteins play a crucial role in pyroptosis, involved in the development and progression of UC. The N-terminal domains of GSDMs, particularly GSDMD and GSDME, form pores in the cell membrane, releasing pro-inflammatory cytokines like IL-1β and IL-18, and other DAMPs that exacerbate inflammation. Importantly, elevations in GSDMD levels have been observed in human intestinal tissues in IBDs [58–61]. Thus, GSDMs in pyroptosis are another therapeutic target in UC. Targeting various stages of GSDM pore formation and its cleavage and activation, oligomerization, membrane insertion, and interaction with cellular components, has been investigated. Compounds that have been demonstrated to inhibit the oligomerization and pore formation of GSDM include necrosulfonamide (NSA), which blocks GSDMD oligomerization by interacting with the C191 amino acid residue [62, 63]. Disulfiram (DSF), an FDA-approved drug for treating alcoholism, inhibits GSDMD by covalently modifying Cys191, preventing the formation of pores and the release of inflammatory factors [62]. Studies in mice with DSS-induced UC found that DSF combined with copper could reverse symptoms and inhibit colonic macrophage activation and reduce inflammation by blocking the NF-κB pathway and decreasing pro-inflammatory cytokine secretion [63]. In addition, DSF combined with copper also protected the intestinal barrier by reversing tight junction protein expression and improved intestinal microflora by reducing harmful bacteria and increasing beneficial bacteria [64]. Dimethyl Fumarate (DMF), an Nrf2 activator used in treating multiple sclerosis, causes GSDMD succination at specific cysteine residues, hindering its interaction with caspases and reducing oligomerization and pyroptosis induction [65]. GI-Y1, a novel GSDMD inhibitor that targets the Arg7 residue of GSDMD, inhibiting lipid binding, pore formation, and mitochondrial binding [66].

### Compounds target NF-kB-mediated inflammatory response and pyroptosis

Bay 11–7082 has been shown to be a NF-κB inhibitor. It also functions as an inhibitor of GSDMD oligomerization by binding to the C191 residue, similar to NSA and DSF [67]. Honokiol is another compound that alleviates UC by targeting the NF-κB signaling pathway and suppressing GSDMD-mediated pyroptosis in vivo and in vitro [68]. Furthermore, micro RNAs (miRNAs) are a class of endogenous, small noncoding RNAs that can promote downregulation of protein expression by translational repression and/or mRNA degradation of target mRNAs involved in inflammation. MiR-124 is a crucial modulator of inflammation and innate immunity that could provide therapeutic restitution of physiological pathways lost in inflammatory diseases [69]. A small quinoline, ABX464 (obefazimod), has been shown to upregulate miR-124 in human immune cells. In a proof-of-concept clinical study, ABX464 showed robust and consistent efficacy in UC [70]. Vermeire et al. [71] recently reported the results from a phase 2b, double-blind, randomised, placebo-controlled induction trial and 48 week, open-label extension of ABX464 in patients with moderate-to-severe ulcerative colitis. Patients were randomly assigned to daily oral ABX464 25 mg (n = 63), 50 mg (n = 63), 100 mg (n = 64), or placebo (n = 64). At week 8, the modified Mayo Score was significantly improved from baseline in all ABX464 dosage groups compared with placebo. Secondary endpoints, such as clinical response, clinical remission, endoscopic improvement, FCP concentration reduction, and histological improvement supported the efficacy of ABX464. Overall, ABX464 was well tolerated.

Repurposing existing medications presents a unique opportunity to enhance the treatment of UC. Drugs such as troxerutin, diacetylrhein, and topiramate have demonstrated promising results in preclinical models of IBD, targeting pivotal pathways including inflammation, oxidative stress, and pyroptosis [72]. These medications hold the potential to provide safer, more targeted, and effective alternatives to conventional therapies, such as TNF and IL-33 therapeutics. For instance, troxerutin exhibits anti-inflammatory and anti-apoptotic properties. In animal models of colitis, troxerutin administration resulted in diminished inflammation and tissue damage, suggesting its potential to alleviate UC symptoms [73]. By integrating these repurposed treatments with biomarkers of pyroptosis, physicians may develop innovative strategies that not only enhance symptom management but also effectively manage the disease while minimizing adverse effects [73, 74]

### Translational perspectives

While current experimental evidence supports a correlation between specific biomarkers and intestinal inflammation in UC, the data remain insufficient to unequivocally establish their exclusive roles in pyroptosis. The majority of studies conducted to date have been conducted with limited patient cohorts or preclinical models, thereby restricting the generalizability of findings to broader patient populations.

To effectively translate these insights into clinical practice, further research is imperative. Large-scale multicenter clinical trials are necessary to validate biomarkers such as CP, lactoferrin, IL-1β, and IL-18, specifically for identifying pyroptosis-driven inflammation in diverse UC patient populations. Longitudinal studies could provide valuable insights by monitoring these biomarkers over time and correlating changes with clinical outcomes, thereby elucidating their predictive value regarding disease progression and therapeutic response. Mechanistic studies in UC should prioritize elucidating the precise molecular pathways involved in pyroptosis. Furthermore, it is crucial to explore interactions with other inflammatory cell death modalities, such as necroptosis and ferroptosis. Interventional trials targeting inflammasome activation or the pyroptotic pathway through inhibitors of NLRP3 or GSDM hold promise in establishing causative links and paving the way for innovative therapeutic strategies.

UC management encompasses a comprehensive approach that integrates pharmacological therapies with lifestyle modifications. These include dietary adjustments [75], regular exercise, and stress management techniques. As future research validates the clinical utility of identified biomarkers, these discoveries can lead to the development of novel therapeutic paradigms specifically designed to alleviate UC symptoms and enhance the quality of life for patients.

## Conclusions

UC is a chronic and relapsing inflammatory condition of the colon that significantly disrupts mucosal homeostasis and immune regulation. In patients with UC, pyroptosis is a highly inflammatory, caspase-dependent form of cell death; the pathogenesis of UC has gained increasing attention due to its capacity to exacerbate mucosal injury through the release of pro-inflammatory cytokines such as IL-1β and IL-18. Dysbiosis, microbial products, mitochondrial dysfunction, and environmental stressors are key triggers of inflammasome activation in colonic epithelial cells, leading to pyroptotic cell death and sustained inflammation. Several biomarkers including PCSK9, GSDM, CRP, calprotectin, and IL-33 have shown diagnostic, prognostic, and therapeutic potential in characterizing UC disease activity and guiding clinical interventions. The identification of pyroptosis and its associated biomarkers represents a significant advancement in understanding the disease’s underlying mechanisms. These discoveries are highly relevant to the field as they offer novel insights into the inflammatory pathways driving mucosal damage, paving the way for more precise diagnostic tools and targeted therapies. By clarifying the molecular underpinnings of pyroptosis, researchers can develop interventions that go beyond symptom control to address the root causes of inflammation, ultimately improving disease management and patient outcomes.

Moving forward, the identification of pyroptosis-associated pathways opens new therapeutic avenues, including the use of inflammasome inhibitors, GSDM-targeting agents, and repurposed medications that modulate oxidative stress and mucosal immunity. However, despite promising preclinical findings, translational gaps remain. Large-scale, longitudinal studies and mechanistic investigations are necessary to confirm the clinical utility of these biomarkers and elucidate their exact roles in UC. Personalized treatment strategies targeting pyroptosis, informed by genetic, microbial, and immunologic profiles, could transform UC management. By integrating biomarker-driven diagnostics with tailored pharmacologic and lifestyle interventions, clinicians and researchers together may significantly improve outcomes and quality of life for individuals living with UC.

## Data Availability

Not applicable.

## Abbreviations

CCL: CC Chemokine ligand
CP: Calprotectin
CXCL: CXC Chemokine ligand
CECs: Colorectal epithelial cells
CD: Crohn’s disease
FCP: Fecal calprotectin
FL: Fecal lactoferrin
GSDM: Gasdermin
IBD: Inflammatory Bowel Disease
IL: Interleukin
MES: Mayo endoscopic subscore
PCSK9: Proprotein convertase subtilisin kexin type 9
ROS: Reactive oxygen species
SAA: Serum amyloid A
TNF: Tumor necrosis factor
UC: Ulcerative colitis

## References

[1] Berre CL, Honap S, Peyrin-Biroulet L. Ulcerative colitis. Lancet. 2023;12(10401):571–84. 10.1016/S0140-6736(23)00966-2. 10.1016/S0140-6736(23)00966-2 37573077

[2] Rubin DT, Ananthakrishnan AN, Siegel CA, Sauer BG, Long MD. ACG clinical guideline in adults. Am J Gastroenterol. 2019;114(3):384–413. 10.14309/ajg.0000000000000152. 30840605 10.14309/ajg.0000000000000152

[3] Gajendran M, Loganathan P, Jimenez G, Catinella AP, Ng N, Umapathy C, et al. A comprehensive review and update on ulcerative colitis. Dis Mon. 2019. 10.1016/j.disamonth.2019.02.004. 30837080 10.1016/j.disamonth.2019.02.004

[4] Zhou Q, Shen ZF, Wu BS, Xu CB, He ZQ, Chen T, et al. Risk of colorectal cancer in ulcerative colitis patients: a systematic review and meta-analysis. Gastroenterol Res Pract. 2019;3:5363261. 10.1155/2019/5363261. 10.1155/2019/5363261 PMC6874962 31781191

[5] Zhang T, Zhang B, Tian W, Wang F, Zhang J, Ma X, et al. Research trends in ulcerative colitis: a bibliometric and visualized study from 2011 to 2021. Front Pharmacol. 2022;13:951004. 10.3389/fphar.2022.951004. 36199683 10.3389/fphar.2022.951004 PMC9529236

[6] Tan G, Huang C, Chen J, et al. HMGB1 released from GSDME-mediated pyroptotic epithelial cells participates in the tumorigenesis of colitis-associated colorectal cancer through the ERK1/2 pathway. J Hematol Oncol. 2020;13:149. 10.1186/s13045-020-00985-0. 33160389 10.1186/s13045-020-00985-0 PMC7648939

[7] Patankar JV, Becker C. Cell death in the gut epithelium and implications for chronic inflammation. Nat Rev Gastroenterol Hepatol. 2020;17:543–56. 10.1038/s41575-020-0326-4. 32651553 10.1038/s41575-020-0326-4

[8] Ai Y, Meng Y, Yan B, Zhou Q, Wang X. The biochemical pathways of apoptotic, necroptotic, pyroptotic, and ferroptotic cell death. Mol Cell. 2024;84(1):170–9. 10.1016/j.molcel.2023.11.040. 38181758 10.1016/j.molcel.2023.11.040

[9] Yu P, Zhang X, Liu N, et al. Pyroptosis: mechanisms and diseases. Sig Transduct Target Ther. 2021;6(1):128. 10.1038/s41392-021-00507-5. 10.1038/s41392-021-00507-5 PMC8005494 33776057

[10] Martinon F, Burns K, Tschopp J. The inflammasome. Mol Cell. 2002;10(2):417–26. 10.1016/S1097-2765(02)00599-3. 12191486 10.1016/s1097-2765(02)00599-3

[11] Shi J, Zhao Y, Wang K, et al. Cleavage of GSDMD by inflammatory caspases determines pyroptotic cell death. Nature. 2015;526:660–5. 10.1038/nature15514. 26375003 10.1038/nature15514

[12] Soderman J, Berglind L, Almer S. Gene expression-genotype analysis implicates GSDMA, GSDMB, and LRRC3C as contributors to inflammatory bowel disease susceptibility. BioMed Res Int. 2015;2015(1):834805. 10.1155/2015/834805. 26484354 10.1155/2015/834805 PMC4592899

[13] Zhao Y, Ma Y, Pei J, et al. Exploring pyroptosis-related signature genes and potential drugs in ulcerative colitis by transcriptome data and animal experimental validation. Inflammation. 2024;47(6):2057–76. 38656456 10.1007/s10753-024-02025-2

[14] Yang D, He Y, Muñoz-Planillo R, Liu Q, Núñez G. Caspase-11 requires the pannexin-1 channel and the purinergic P2X7 pore to mediate pyroptosis and endotoxic shock. Immunity. 2015;43:923–32. 10.1016/j.immuni.2015.10.009. 26572062 10.1016/j.immuni.2015.10.009 PMC4795157

[15] Bauer C, Duewell P, Lehr H, Endres S, Schnurr M. Protective and aggravating effects of Nlrp3 inflammasome activation in IBD models: influence of genetic and environmental factors. Dig Dis. 2012;30(1):82–90. 10.1159/000341681. 23075874 10.1159/000341681

[16] Mortlock S, Lord A, Montgomery G, Zakrzewski M, Simms LA, Krishnaprasad K, et al. An extremes of phenotype approach confirms significant genetic heterogeneity in patients with ulcerative colitis. J Crohn’s Colitis. 2023;17(2):277–88. 36111848 10.1093/ecco-jcc/jjac121 PMC10024548

[17] Wei D, Guo H, Xu C, Wang B, Zhang M, Ding F. Mitochondrial reactive oxygen species-mediated NLRP3 inflammasome activation contributes to aldosterone-induced renal tubular cell injury. Oncotarget. 2016;7:17479–91. 27014913 10.18632/oncotarget.8243 PMC4951227

[18] Ghosh S, Nukavarapu S, Jala VR. Effects of heavy metals on gut barrier integrity and gut microbiota. Microb Host. 2024. 10.1530/MAH-23-0015.

[19] De Santis S, Cavalcanti E, Mastronardi M, Jirillo E, Chieppa M. Nutritional keys for intestinal barrier modulation. Front Immunol. 2015;6:612. 26697008 10.3389/fimmu.2015.00612 PMC4670985

[20] Chen K, Shang S, Yu S, et al. Identification and exploration of pharmacological pyroptosis-related biomarkers of ulcerative colitis. Front Immunol. 2022;13:998470. 36311726 10.3389/fimmu.2022.998470 PMC9606687

[21] Ning Y, Lin K, Fang J, et al. Pyroptosis-related signature predicts the progression of ulcerative colitis and colitis-associated colorectal cancer as well as the anti-TNF therapeutic response. J Immunol Res. 2023;2023:7040113. 36741232 10.1155/2023/7040113 PMC9897931

[22] Lei-Leston AC, Murphy AG, Maloy KJ. Epithelial cell inflammasomes in intestinal immunity and inflammation. Front Immunol. 2017;8:1168. 10.3389/fimmu.2017.01168. 28979266 10.3389/fimmu.2017.01168 PMC5611393

[23] Gilliland A, Chan JJ, De Wolfe TJ, Yang H, Vallance BA. Pathobionts in inflammatory bowel disease: origins, underlying mechanisms, and implications for clinical care. Gastroenterology. 2024;166(1):44–58. 10.1053/j.gastro.2023.09.019. 37734419 10.1053/j.gastro.2023.09.019

[24] Sasso JM, Ammar RM, Tenchov R, et al. Gut microbiome-brain alliance: a landscape view into mental and gastrointestinal health and disorders. ACS Chem Neurosci. 2023;14(10):1717–63. 10.1021/acschemneuro.3c00127. 37156006 10.1021/acschemneuro.3c00127 PMC10197139

[25] Shen ZH, Zhu CX, Quan YS, et al. Relationship between intestinal microbiota and ulcerative colitis: mechanisms and clinical application of probiotics and fecal microbiota transplantation. World J Gastroenterol. 2018;24(1):5–14. 10.3748/wjg.v24.i1.5. 29358877 10.3748/wjg.v24.i1.5 PMC5757125

[26] Zheng M, Han R, Yuan Y, et al. The role of *Akkermansia muciniphila* in inflammatory bowel disease: current knowledge and perspectives. Front Immunol. 2023;13:1089600. 10.3389/fimmu.2022.1089600. 36685588 10.3389/fimmu.2022.1089600 PMC9853388

[27] Xu X, Huang Z, Huang Z, et al. Butyrate attenuates intestinal inflammation in Crohn’s disease by suppressing pyroptosis of intestinal epithelial cells via the cGSA-STING-NLRP3 axis. Int Immunopharmacol. 2024;143(2):113305. 10.1016/j.intimp.2024.113305. 39426229 10.1016/j.intimp.2024.113305

[28] Tontini GE, Rath T, Neumann H. Advanced gastrointestinal endoscopic imaging for inflammatory bowel diseases. World J Gastroenterol. 2016;22(3):1246–59. 10.3748/wjg.v22.i3.1246. 26811662 10.3748/wjg.v22.i3.1246 PMC4716035

[29] Wangchuk P, Yeshi K, Loukas A. Ulcerative colitis: clinical biomarkers, therapeutic targets, and emerging treatments. Trends Pharmacol Sci. 2024;45(10):892–903. 10.1016/j.tips.2024.08.003. 39261229 10.1016/j.tips.2024.08.003

[30] Guardiola J, Lobatón T, Cerrillo E, Ferreiro-Iglesias R, Gisbert JP, Domènech E, et al. Recommendations of the Spanish working group on Crohn’s disease and ulcerative colitis (GETECCU) on the utility of the determination of faecal calprotectin in inflammatory bowel disease. Gastroenterol Hepatol. 2018;41(8):514–29. 10.1016/j.gastrohep.2018.05.029. 30293556 10.1016/j.gastrohep.2018.05.029

[31] Wang X, Li X, Liu S, et al. Pcsk9 regulates pyroptosis via mtdna damage in chronic myocardial ischemia. Basic Res Cardiol. 2020;115(6):66. 10.1007/s00395-020-00832-w. 33180196 10.1007/s00395-020-00832-w

[32] Yang J, Ma X, Niu D, Sun Y, Chai X, Deng Y, et al. PCSK9 inhibitors suppress oxidative stress and inflammation in atherosclerotic development by promoting macrophage autophagy. Am J Transl Res. 2023;15(8):5129–44. 37692938 PMC10492065

[33] Marinelli C, Zingone F, Lupo MG, et al. Serum levels of PCSK9 are increased in patients with active ulcerative colitis representing a potential biomarker of disease activity: a cross-sectional study. J Clin Gastroenterol. 2022;56(9):787–93. 10.1097/MCG.0000000000001607. 34560758 10.1097/MCG.0000000000001607 PMC9988229

[34] Wiercinska-Drapalo A, Flisiak R, Jaroszewicz J, Prokopowicz D. Plasma interleukin-18 reflects severity of ulcerative colitis. World J Gastroenterol. 2005;11(4):605–8. 10.3748/wjg.v11.i4.605. 15641156 10.3748/wjg.v11.i4.605 PMC4250821

[35] Nowarski R, Jackson R, Gagliani N, de Zoete MR, Palm NW, Bailis W, et al. Epithelial IL-18 equilibrium controls barrier function in colitis. Cell. 2015;163(6):1444–56. 10.1016/j.cell.2015.10.072. 26638073 10.1016/j.cell.2015.10.072 PMC4943028

[36] Kobori A, Yagi Y, Imaeda H, Ban H, Bamba S, Tsujikawa T, et al. Interleukin-33 expression is specifically enhanced in inflamed mucosa of ulcerative colitis. J Gastroenterol. 2010;45:999–1007. 10.1007/s00535-010-0245-1. 20405148 10.1007/s00535-010-0245-1

[37] Pastorelli L, Garg RR, Hoang SB, Spina L, Mattioli B, Scarpa M, et al. Epithelial-derived IL-33 and its receptor ST2 are dysregulated in ulcerative colitis and in experimental Th1/Th2 driven enteritis. Proc Natl Acad Sci USA. 2010;107:8017–22. 10.1073/pnas.0912678107. 20385815 10.1073/pnas.0912678107 PMC2867895

[38] Chauvin C, Retnakumar SV, Bayry J. Gasdermin D as a cellular switch to orientate immune responses via IL-33 or IL-1β. Cell Mol Immunol. 2023;20(1):8–10. 10.1038/s41423-022-00950-6. 36380096 10.1038/s41423-022-00950-6 PMC9664042

[39] Sproston NR, Ashworth JJ. Role of C-reactive protein at sites of inflammation and infection. Front Immunol. 2018;9:754. 10.3389/fimmu.2018.00754. 29706967 10.3389/fimmu.2018.00754 PMC5908901

[40] Rajab IM, Hart PC, Potempa LA. How C-reactive protein structural isoforms with distinctive bioactivities affect disease progression. Front Immunol. 2020;11:2126. 10.3389/fimmu.2020.02126. 33013897 10.3389/fimmu.2020.02126 PMC7511658

[41] Grant RK, Jones GR, Plevris N, Lynch RW, Jenkinson PW, Lees CW, et al. The ACE (albumin, CRP and endoscopy) index in acute colitis: a simple clinical index on admission that predicts outcome in patients with acute ulcerative colitis. Inflamm Bowel Dis. 2021;27(4):451–7. 10.1093/ibd/izaa088. 32572468 10.1093/ibd/izaa088

[42] Wakai M, Hayashi R, Tanaka S, et al. Serum amyloid A is a better predictive biomarker of mucosal healing than C-reactive protein in ulcerative colitis in clinical remission. BMC Gastroenterol. 2020;20:85. 10.1186/s12876-020-01229-8. 32245401 10.1186/s12876-020-01229-8 PMC7118889

[43] Walsham N, Sherwood R. Fecal calprotectin in inflammatory bowel disease. Clin Exp Gastroenterol. 2016;9:21–9. 10.2147/CEG.S51902. 26869808 10.2147/CEG.S51902 PMC4734737

[44] Zhang S, Liang Y, Yao J, Li D, Wang L. Role of pyroptosis in inflammatory bowel disease (IBD): from gasdermins to DAMPs. Front Pharmacol. 2022;13:833588. 10.3389/fphar.2022.833588. 35677444 10.3389/fphar.2022.833588 PMC9168461

[45] Heinzel S, Jureczek J, Kainulainen V, Nieminen A, Suenkel U, Thaler A, et al. Elevated fecal calprotectin is associated with gut microbial dysbiosis, altered serum markers and clinical outcomes in older individuals. Sci Rep. 2024;14:13513. 10.1038/s41598-024-63893-0. 38866914 10.1038/s41598-024-63893-0 PMC11169261

[46] Kane SV, Sandborn WJ, Rufo PA, et al. Fecal lactoferrin is a sensitive and specific marker in identifying intestinal inflammation. Am J Gastroenterol. 2003;98(6):1309–14. 10.1111/j.1572-0241.2003.07458.x. 12818275 10.1111/j.1572-0241.2003.07458.x

[47] Sorrentino D, Nguyen VQ, Love K. Fecal lactoferrin predicts primary nonresponse to biologic agents in inflammatory bowel disease. Dig Dis. 2021;39(6):626–33. 10.1159/000515432. 33631768 10.1159/000515432 PMC8686729

[48] Rubio MG, Amo-Mensah K, Gray JM, Nguyen VQ, Nakat S, Grider D, et al. Fecal lactoferrin accurately reflects mucosal inflammation in inflammatory bowel disease. World J Gastrointest Pathophysiol. 2019;10(5):54–63. 10.4291/wjgp.v10.i5.54. 31911845 10.4291/wjgp.v10.i5.54 PMC6940564

[49] Zhu C, Xu S, Jiang R, et al. The gasdermin family: emerging therapeutic targets in diseases. Sig Transduct Target Ther. 2024;9:87. 10.1038/s41392-024-01801-8. 10.1038/s41392-024-01801-8 PMC10999458 38584157

[50] Burdette BE, Esparza AN, Zhu H, Wang S. Gasdermin D in pyroptosis. Acta Pharm Sin B. 2021;11(9):2768–82. 10.1016/j.apsb.2021.02.006. 34589396 10.1016/j.apsb.2021.02.006 PMC8463274

[51] Yuan Y, Xie K, Wang S, Yuan L. Inflammatory caspase-related pyroptosis: mechanism, regulation, and therapeutic potential for inflammatory bowel disease. Gastroenterol Rep. 2018;6(3):167–76. 10.1093/gastro/goy011. 10.1093/gastro/goy011 PMC6101557 30151200

[52] Ma F, Ghimire L, Ren Q, et al. Gasdermin E dictates inflammatory responses by controlling the mode of neutrophil death. Nat Commun. 2024;15:386. 10.1038/s41467-023-44669-y. 38195694 10.1038/s41467-023-44669-y PMC10776763

[53] Korta A, Kula J, Gomułka K. The Role of IL-23 in the Pathogenesis and Therapy of Inflammatory Bowel Disease. Int J Mol Sci. 2023;24(12):10172. 10.3390/ijms241210172. 37373318 10.3390/ijms241210172 PMC10299037

[54] Sewell GW, Kaser A. Interleukin-23 in the Pathogenesis of Inflammatory Bowel Disease and Implications for Therapeutic Intervention. J Crohn Colitis. 2022. 10.1093/ecco-jcc/jjac034. 10.1093/ecco-jcc/jjac034 PMC9097674 35553667

[55] Viola A, Pugliese D, Renna S, Furfaro F, Caprioli F, D’Incà R, et al. Outcome in ulcerative colitis after switch from adalimumab/golimumab to infliximab: a multicenter retrospective study. Dig Liver Dis. 2019;51(4):510–5. 10.1016/j.dld.2018.10.013. 30472389 10.1016/j.dld.2018.10.013

[56] Wang L, Chen P, He S, Duan S, Zhang Y. Predictors and optimal management of tumor necrosis factor antagonist nonresponse in inflammatory bowel disease: a literature review. World J Gastroenterol. 2023;29(29):4481–98. 10.3748/wjg.v29.i29.4481. 37621757 10.3748/wjg.v29.i29.4481 PMC10445007

[57] Konikoff MR, Denson LA. Role of fecal calprotectin as a biomarker of intestinal inflammation in inflammatory bowel disease. Inflamm Bowel Dis. 2006;12(6):524–34. 10.1097/00054725-200606000-00013. 16775498 10.1097/00054725-200606000-00013

[58] de Vasconcelos NM, Lamkanfi M. Recent insights on inflammasomes, Gasdermin pores, and pyroptosis. Cold Spring Harb Perspect Biol. 2020;12(5):a036392. 10.1101/cshperspect.a036392. 31570336 10.1101/cshperspect.a036392 PMC7197430

[59] Liu X, Zhang Z, Ruan J, Pan Y, Magupalli VG, Wu H, et al. Inflammasome-activated gasdermin D causes pyroptosis by forming membrane pores. Nature. 2016;535:153–215. 10.1038/nature18629. 27383986 10.1038/nature18629 PMC5539988

[60] Rathkey JK, Zhao J, Liu Z, Chen Y, Yang J, Kondolf HC, et al. Chemical disruption of the pyroptotic pore-forming protein gasdermin D inhibits inflammatory cell death and sepsis. Sci Immunol. 2018. 10.1126/sciimmunol.aat2738. 30143556 10.1126/sciimmunol.aat2738 PMC6462819

[61] Zhou B, Abbott DW. Chemical modulation of gasdermin D activity: therapeutic implications and consequences. Semin Immunol. 2023;70:101845. 10.1016/j.smim.2023.101845. 37806032 10.1016/j.smim.2023.101845 PMC10841450

[62] Hu JJ, Liu X, Xia S, Zhang Z, Zhang Y, Zhao J, et al. FDA-approved disulfiram inhibits pyroptosis by blocking gasdermin D pore formation. Nat Immunol. 2020. 10.1038/s41590-020-0669-6. 32367036 10.1038/s41590-020-0669-6 PMC7316630

[63] Zhou W, Zhang H, Huang L, Sun C, Yue Y, Cao X, et al. Disulfiram with Cu2+ alleviates dextran sulfate sodium-induced ulcerative colitis in mice. Theranostics. 2023;13(9):2879–95. 10.7150/thno.81571. 37284442 10.7150/thno.81571 PMC10240830

[64] Hu H, Cui L, Lu J, Wei K, Wei J, Li S, et al. Intestinal microbiota regulates anti-tumor effect of disulfiram combined with Cu2+ in a mice model. Cancer Med. 2020;9(18):6791–801. 10.1002/cam4.3346. 32750218 10.1002/cam4.3346 PMC7520343

[65] Humphries F, Shmuel-Galia L, Ketelut-Carneiro N, Li S, Wang B, Nemmara VV, et al. Succination inactivates gasdermin D and blocks pyroptosis. Science. 1979;369:1633–7. 10.1126/science.abb9818. 10.1126/science.abb9818 PMC8744141 32820063

[66] Fan X, Cheng Z, Shao R, Ye K, Chen X, Cai X, et al. The novel GSDMD inhibitor GI-Y2 exerts antipyroptotic effects to reduce atherosclerosis. Clin Transl Med. 2025;15(3):e70263. 10.1002/ctm2.70263. 40045452 10.1002/ctm2.70263 PMC11882392

[67] Pandeya A, Li L, Li Z, Wei Y. Gasdermin D (GSDMD) as a new target for the treatment of infection. Medchemcomm. 2019;10(5):660–7. 10.1039/c9md00059c. 31191857 10.1039/c9md00059c PMC6533889

[68] Wang N, Kong R, Han W, Bao W, Shi Y, Ye L, et al. Honokiol alleviates ulcerative colitis by targeting PPAR-γ-TLR4-NF-κB signaling and suppressing gasdermin-D-mediated pyroptosis in vivo and in vitro. Int Immunopharmacol. 2022;111:109058. 10.1016/j.intimp.2022.109058. 35901530 10.1016/j.intimp.2022.109058

[69] Yuan W, Tian Y, Lin C, Wang Y, Liu Z, Zhao Y, et al. Pectic polysaccharides derived from Hainan Rauwolfia ameliorate NLR family pyrin domain-containing 3-mediated colonic epithelial cell pyroptosis in ulcerative colitis. Physiol Genomics. 2023;55(1):27–40. 10.1152/physiolgenomics.00081.2022. 36440907 10.1152/physiolgenomics.00081.2022

[70] Apolit C, Campos N, Vautrin A, Begon-Pescia C, Lapasset L, Scherrer D, et al. ABX464 (Obefazimod) Upregulates miR-124 to Reduce Proinflammatory Markers in Inflammatory Bowel Diseases. Clin Transl Gastroenterol. 2023;14(4):e00560. 10.14309/ctg.0000000000000560. 36573890 10.14309/ctg.0000000000000560 PMC10132720

[71] Vermeire S, Sands BE, Tilg H, Tulassay Z, Kempinski R, Danese S, et al. ABX464 (obefazimod) for moderate-to-severe, active ulcerative colitis: a phase 2b, double-blind, randomised, placebo-controlled induction trial and 48 week, open-label extension. Lancet Gastroenterol Hepatol. 2022;7(11):1024–35. 10.1016/S2468-1253(22)00233-3. 36075249 10.1016/S2468-1253(22)00233-3

[72] Bhat MA, Usman I, Dhaneshwar S. Application of drug repurposing approach for therapeutic intervention of inflammatory bowel disease. Curr Rev Clin Exp Pharmacol. 2024;19(3):234–49. 10.2174/0127724328245156231008154045. 37859409 10.2174/0127724328245156231008154045

[73] Wang X, Gao Y, Wang L, Yang D, Bu W, Gou L, et al. Troxerutin improves dextran sulfate sodium-induced ulcerative colitis in mice. J Agric Food Chem. 2021;69(9):2729–44. 10.1021/acs.jafc.0c06755. 33621077 10.1021/acs.jafc.0c06755

[74] Coll RC, Schroder K, Pelegrín P. Nlrp3 and pyroptosis blockers for treating inflammatory diseases. Trends Pharmacol Sci. 2022;43(8):653–68. 10.1016/j.tips.2022.04.003. 35513901 10.1016/j.tips.2022.04.003

[75] Lu X, Sun Y, Zhang Z, Sun Z, Wang S, Xu E. Regulation of pyroptosis by natural products in ulcerative colitis: mechanisms and therapeutic potential. Front Pharmacol. 2025;9(16):1573684. 10.3389/fphar.2025.1573684. 10.3389/fphar.2025.1573684 PMC12014637 40271055

